# A Comparative Study of Serum Uric Acid levels and Lipid Ratios in Coronary Artery Disease Patients

**Published:** 2014-06

**Authors:** R. Sathiya, V. Kuzhandai Velu, G. Niranjan, A. R. Srinivasan, Ganesh B. Amirtha, R. Ramesh, M. Sathish Babu, Subiman Saha

**Affiliations:** 1 Department of Biochemistry, Mahatma Gandhi Medical College and Research Institute (SBV), Pillaiyarkuppam, Puducherry, India;; 2 Department of Cardiology, Mahatma Gandhi Medical College and Research Institute (SBV), Pillaiyarkuppam, Puducherry, India

**Keywords:** Coronary Artery Disease, Serum Uric Acid, Lipid Ratio

## Abstract

**Introduction:**

Coronary Artery Disease (CAD) appears to be common in the Indian population of different geographical origins, religions and languages. Measurement of lipid fractions and ratios are widely recommended for risk assessment. A few studies have shown that serum uric acid plays a role in the development of cardiovascular morbidity. Very few reports are cited linking serum uric acid with the lipid fraction in CAD

**Objectives:**

To find the significance of non-HDL cholesterol, LDL-c/HDL-c ratio, TC/HDL ratio and serum uric acid level in CAD patients

**Subjects and Methodology:**

In this study, we included fifty CAD patients as subjects and an equal number of controls. Both subjects and controls were assessed for anthropometric, physiological and biochemical parameters

**Results:**

The present study showed significant increased levels of total cholesterol (*p*=0.002), TAGs (*p*<0.001), HDL (*p*=0.005), LDL (*p*<0.006) and non-HDL cholesterol (*p*<0.001). LDL-c/HDL-c ratio (*p*<0.001) and TC/HDL ratio (*p*<0.001) in CAD patients (subjects) were also significant when compared to controls. Uric acid level in CAD patients was increased (*p*<0.001).

**Conclusion:**

Serum Uric Acid, TC/HDL and LDL/HDL ratios could be regarded as objective markers, in association with existing atherogenic dyslipidemia in patients with CAD.

## INTRODUCTION

The cardiovascular diseases (CVD) account for one-third of all deaths worldwide, two thirds of which occur in the developing countries like India (1). It has been estimated that there will be a doubling of deaths attributed to CVD in India from 1985 to 2015. CAD is defined as a reduction in internal diameter ≥50%, of at least one major coronary artery branch. Measurement of Total Cholesterol (TC), Low Density Lipoprotein (LDL) cholesterol, and High Density Lipoprotein (HDL) cholesterol are widely recommended for CAD risk assessment. Studies have shown that the estimation of Non-HDL cholesterol (Non-HDL) including all other atherogenic factors such as dyslipidemia, apolipoprotein B containing lipoproteins are simpler and better screening tools for assessment of CAD risk in adults (2, 3). Dyslipidemia has been long recognized as a major biochemical event predisposing to atherogenicity. LDL/HDL ratio (LDL/HDL) and total cholesterol/HDL ratio (TC/HDL), which can be obtained from a standard lipid profile is more accurate than either LDL or HDL alone and has also proved to be an accurate predictor of cardiovascular risk factors (5, 6).

Studies citing serum uric acid as an independent marker for CAD are available (7-9). More than five decades ago, *Gertler* and colleagues had postulated that an increase in uric acid levels is a risk factor for CAD (10). A number of studies have shown that serum uric acid plays a role in predicting cardiovascular morbidity (11). The positive association between serum uric acid and cardiovascular diseases(CVD) has been well recognized by several epidemiological studies (8). But, the exact role of uric acid in predicting cardiovascular mortality is still a matter of debate as many studies suggest that hyperuricaemia is associated with CVD because of confounding factors such as obesity, hypertension, use of diuretics and insulin resistance (6, 7).  This study was undertaken to evaluate whether there exist any significant difference in serum uric acid, LDL/HDL and TC/HDL ratio between CAD patients and apparently healthy adults.

## SUBJECTS & METHODOLOGY

Data was collected from fifty CAD patients as subjects and an equal number of controls based on the inclusion and exclusion criteria. The study was conducted at Mahatma Gandhi Medical College & Research Institute (MGMC & RI), a tertiary healthcare institution in Puducherry, after obtaining clearance from institutional human ethics committee.

### Inclusion criteria

- Newly diagnosed CAD patients who had attended Cardiology OPD and General Medicine OPD and as confirmed by angiography.
- Healthy volunteers (Employed in MGMC&RI [for controls] at the point of time).

### Exclusion criteria

- Patients with endocrinological disorders
- Patients who are already on diuretic, antihypertensive, hypolipidemic and anti-gout therapy

A standardized questionnaire comprehensively covering the present and past medical history of CAD (subjects) and controls was also enabled.

### Anthropometric measurements

The height and weight were measured (without shoes), using standard apparatus. Weight was measured to the nearest 0.1 kg and height was measured to the nearest 0.5 cm. Waist circumference was measured to the nearest millimetre at the point midway between the iliac crest and the costal margin. The body mass index (BMI) was calculated using weight (kg)/Height (m^2^).

### Sample collection

Venous blood (5 ml) was drawn from the anterior cubital vein following overnight fast of 10-12 hours. Serum was separated for analysing biochemical parameters.

### Biochemical parameters

Total cholesterol, Triacylglycerols, High density lipoprotein cholesterol (HDL), Low density lipoprotein cholesterol (LDL) and Serum Uric acid were analysed by auto-analyser and IFCC approved methods.

### Statistical Analysis

To compare the means of subjects and controls unpaired Student’t test was used by using SPSS 20 version software.

## RESULTS

The mean and standard deviation of anthropometric and physiological measures of controls and subjects are depicted in Table 1.

**Table 1:** Anthropometric and physiological measures of controls and subjects (CAD)

Parameter	Controls (Mean ± SD)	Subjects (Mean ± SD)	*p* -value

W.C (cm)^a^	39.68 ± 4.31	42.62 ± 7.71	0.020
SBP (mmHg)^b^	131.5 ± 19.55	140.3 ± 17.9	0.000
DBP (mmHg)^a^	73.96 ± 9.56	91.56 ± 13.3	0.020

a *p*<0.05

b *p*<0.005

Data expressed as mean ± SD. W.C, Waist Circumference; SBP, Systolic Blood Pressure; DBP, Diastolic Blood Pressure.

### Fasting lipid profile levels in controls and CAD patients

Table 2 shows the comparison of mean, standard deviation of fasting lipid profile between controls and subjects. This study showed the significance of total cholesterol, low density lipoprotein (LDL), high density lipoprotein (HDL) and triacylglycerol (TAGs).

**Table 2:** Select Biochemical parameters in controls and subjects (CAD)

Parameter	Controls (Mean ± SD)	Subjects (Mean ± SD)	*p*-value

Total cholesterol (mg/dl)^b^	155.48 ± 24.68	181.68 ± 52.43	0.002
TAGs (mg/dl)^b^	100.48 ± 27.04	130.34 ± 44.93	0.000
HDL (mg/dl)^a^	41.91 ± 6.92	38.02 ± 6.65	0.005
LDL (mg/dl)^b^	104.72 ± 24.72	122.10 ± 35.66	0.006
Non-HDL (mg/dl)^b^	113.57 ± 22.54	139.66 ± 51.25	0.000
LDL/HDL^b^	2.60 ± 1.02	2.89 ± 0.99	0.003
TC/HDL^b^	3.79 ± 0.77	4.44 ± 1.54	0.000
Uric acid(mg/dl)^§^	4.35 ± 1.21	5.59 ± 1.29	0.000

a *p*<0.05

b *p*<0.005

Data expressed as mean ± SD. T.C, Total Cholesterol; TAGs, Triacylglycerols; HDL, High Density Lipoprotein; LDL, Low Density Lipoprotein

### Calculated parameters of lipid profile levels in controls and CAD patients

The results in Table 2 show mean and standard deviation of Non-HDL Cholesterol, LDL/HDL ratio and TC/HDL ratio between controls and subjects. From the study, it’s clear that LDL/HDL ratio, TC/HDL ratio and Non-HDL Cholesterol have significant difference between Subjects (CAD patients) and Controls group.

### Fasting serum uric acid level in controls and CAD patients

Table 2 depicts the mean and standard deviation of serum uric acid levels (fasting) between controls and subjects. The study depicted a significant difference in serum uric acid levels among CAD patients and controls.

## DISCUSSION

Several well known risk factors are established for CVD, which can be grouped in to modifiable and non-modifiable. Atherogenic dyslipidemia, include high LDL-C, VLDL and TAG levels with low HDL-C levels is a modifiable risk in both genders. The contribution of atherogenic dyslipidemia to cardiovascular risk is well established based on several epidemiological studies (51, 1). We noted significant atherogenic dyslipidemia and abnormal lipid ratios in patients with CAD (Table 2). The serum LDL-C concentration in CAD patients was significantly high (*p*<0.001) (Table 2). This was similar to the study by *Hammoudeh et al*. There is a great diversity in the extent of atherosclerosis and in the expression of clinical disease. The so called “oxidation hypothesis” states that the oxidative modification of LDL-C (or other lipoprotein) is important and possibly obligatory in the pathogenesis of the atherosclerotic lesion (17).

In the present study, we noted the decrease in serum HDL-C concentration in the CAD patients (Table 2). Similar studies have been reported that HDL-C levels are decreased in patients with CAD. TC/HDL ratios significantly was higher in familial hypercholesterolaemia associated coronary heart disease (18). Evidence presented that HDL-C is inversely related to total body cholesterol (19).

Low serum concentration of HDL-C is also an important risk factor for CAD. In fact, a subgroup of patients with low total cholesterol level (<200 mg/dl), but low HDL-C levels, (<35 to 40 mg/dl) are still at a high risk for atherosclerosis. Traditional cholesterol measurements tend to be the most accurate at predicting risk in comparison to those at the lower and higher ends of the risk spectrum. These measurements are less helpful in the majority of people whose risk falls somewhere in between. Hence, recently lipid ratios, such as TC/HDL-C, LDL-C/HDL-C and TAG/ HDL-C have gained attention. Changes in these ratios have been shown to be better indicators of successful CAD risk reduction than changes in absolute levels of lipids or lipoproteins (20). The LDL-C/HDL-C and TC/HDL-C ratios help initiating lipid-lowering therapy (3, 15, 16). In the *Helsinki* Study, a five year clinical trial of more than 4,000 middle-aged men with elevated lipids, the LDL-C/HDL-C ratio and TC/HDL-C ratio (18, 19) had more prognostic value (16). The current *NCEP guidelines* recommend levels of LDL-C and HDL-C that represent a ratio of about 2.5. Atherogenic dyslipidemia as indicated by increased LDL-C and decreased HDL-C are highly atherogenic and hence, increased LDL-C/HDL-C ratio would be a feasible way to assess the risk of developing CAD.

This study reports that LDL-C/HDL-C and TC/HDL-C ratios in CAD patients were significantly high compared to controls (Table 2). LDL-C/HDL-C ratio could be a useful tool to assess the risk of complications in CAD and also to monitor the patients who are on treatment. In the present study, there was an increased LDL-C level. A similar study showed that LDL-C/HDL-C ratio and TC/HDL-C ratio also correlate with cardiovascular disease (24). Participants in our study had increased levels of Non-HDL-C in CAD (Table 2) Similarly, Jamal *et al.,* have reported an increased level of non-HDL-C that was an independent risk in patients with CAD (25).

Our study showed an increase in serum uric acid levels in CAD patients that was significant than the control group (Table 2). But, this increase in uric acid concentration was statistically significant (*p*<0.001) as compared to control group. In the current study, we also found positive correlations between serum uric acid and CAD. The Framingham Heart Study and Atherosclerosis in Communities (ARIC) study data failed to disclose an association between uric acid and CAD. Several studies have revealed that uric acid may be associated with the presence of CAD (26). Among those with suspected CAD who had undergone coronary angiography, serum uric acid concentration of greater than 416 µmol/L (7.0 mg/dl) was associated with stable plaques without evidence of remodeling. This suggests that uric acid is a marker of atherosclerosis (27-29). In the present study, an effort was made to study the lipid fractions, *viz* serum total cholesterol, triacylglycerols, LDL, HDL, LDL/HDL ratio, TC/HDL ratio, Non-HDL-C and serum uric acid levels in subjects having CAD. The results were compared with control samples.

## CONCLUSION

Significant increase in serum total cholesterol levels and LDL-C levels were noted in patients with CAD. A decreased HDL-C concentration with high serum uric acid concentration was observed in CAD patients compared to the controls. This facilitates the claim that serum uric acid in association with the lipid ratios could serve as simple and economically viable biochemical marker.
